# Crosstalk between MUC1 and VEGF in angiogenesis and metastasis: a review highlighting roles of the MUC1 with an emphasis on metastatic and angiogenic signaling

**DOI:** 10.1186/s12935-021-01899-8

**Published:** 2021-04-09

**Authors:** Farnaz Khodabakhsh, Parnaz Merikhian, Mohammad Reza Eisavand, Leila Farahmand

**Affiliations:** 1 Department of Genetics and Advanced Medical Technology, Medical Biotechnology Research Center, Faculty of Medicine, AJA University of Medical Sciences, Tehran, Iran; 2 Recombinant Proteins Department, Breast Cancer Research Center, Motamed Cancer Institute, ACECR, No. 146, South Gandhi Ave., Vanak Sq., Tehran, Iran

**Keywords:** Angiogenesis, VEGF, VEGFR, MUC1, Cancer, Targeted therapy

## Abstract

VEGF and its receptor family (VEGFR) members have unique signaling transduction system that play significant roles in most pathological processes, such as angiogenesis in tumor growth and metastasis. VEGF-VEGFR complex is a highly specific mitogen for endothelial cells and any de-regulation of the angiogenic balance implicates directly in endothelial cell proliferation and migration. Moreover, it has been shown that overexpressing Mucin 1 (MUC1) on the surface of many tumor cells resulting in upregulation of numerous signaling transduction cascades, such as growth and survival signaling pathways related to RTKs, loss of cell-cell and cell-matrix adhesion, and EMT. It promotes gene transcription of pro-angiogenic proteins such as HIF-1α during periods of oxygen scarcity (hypoxia) to enhance tumor growth and angiogenesis stimulation. In contrast, the cytoplasmic domain of MUC1 (MUC1-C) inhibits apoptosis, which in turn, impresses upon cell fate. Besides, it has been established that reduction in VEGF expression level correlated with silencing MUC1-C level indicating the anti-angiogenic effect of MUC1 downregulation. This review enumerates the role of MUC1-C oncoprotein and VEGF in angiogenesis and metastasis and describes several signaling pathways by which MUC1-C would mediate the pro-angiogenic activities of cancer cells.

## Background

### Angiogenesis

The vascular system is mainly required for nutrient and oxygen delivery, metabolite waste removal, and immune surveillance. Angiogenesis, the formation of new blood vessels from pre-existing blood vessels, is a multi-step process that biologically plays an essential role in health such as embryonic development, wound healing, and menstruation cycle [1, 2]. Unregulated angiogenesis is similarly observed in pathological conditions including diabetic retinopathy, rheumatoid arthritis, and particularly cancer as a critical factor in tumor development and metastasis [3]. Angiogenesis is induced by tumor cells, allowing them to establish a blood supply system leading to accelerated growth, survival, and invasiveness [4].

For gaining the ability of growth and invasion, malignant cells overexpress angiogenic factors to induce tumor neovascularization and remodel tumor vessels. Additionally, tumor-derived-angiogenic factors and cytokines can be released by various host cells such as inflammatory cells, adjacent stromal fibroblasts, and perivascular cells in the area of tumor microenvironment [4, 5]. Most tumor-derived-angiogenic factors trigger angiogenic signaling through binding to their cell surface receptors which frequently comprise tyrosine kinase domains. Moreover, various angiogenic factors appear to generally have distinct functions in the regulation of vessel growth, vascular permeability, and remodeling. The abundant presence of angiogenic factors and cytokines together inevitably results in the interplay of various intracellular signaling cascades, leading to synergistic effects of tumor angiogenesis [4]. The process of blood vessel angiogenesis and lymphangiogenesis formation is regulated by multiple growth factors and cytokines such as vascular endothelial growth factor (VEGF), basic fibroblast growth factor (b-FGF or FGF-2), angiogenin (Ang), transforming growth factor (TGF-α, TGF-β), platelet-derived endothelial growth factor (PDGF), granulocyte colony-stimulating factor (GCSF), hepatocyte growth factor (HGF), epidermal growth factor (EGF), tumor necrosis factor (TNF-α) and interleukin-1 and 8 [6, 7]. The full performance of angiogenesis necessitates complex signaling formation using VEGF and its receptors (VEGFRs). Among these factors, the VEGF family is the most pro-angiogenic factor in cancer [4].

### Vascular endothelial growth factor (VEGF)

VEGF, also named VEGF-A, is a homodimeric glycoprotein with a molecular weight of approximately 45 kDa [8]. Other isoforms, which include VEGF-B, VEGF-C, VEGF-D, placenta growth factor (PLGF), VEGF-E (Orf-VEGF), and *Trimeresurus flavoviridis* svVEGF show varying degrees of homology with VEGF as a result of alternative patterns of splicing [8]. There are three tyrosine kinase VEGF receptors: VEGFR1 (Flt-1), VEGFR2 (KDR/Flk-1), and VEGFR3 (Flt-4) that their structures are homologous with each other [9]. VEGF-A and its tyrosine kinase receptors, VEGFR-1 (Flt-1) and VEGFR-2 (KDR/Flk-1), have been categorized as one of the main stimulators of oncogenic angiogenesis prompted under hypoxic condition [10, 11]. VEGFR-1 has an extremely strong ligand-binding domain for VEGF-A (Kd =  1–10 pM), compared with VEGFR-2 that has robust tyrosine kinase activity. VEGFR-2 expression is up-regulated in the tumor vasculature compared to the normal vasculature and represents a critical role in the endothelial cells (ECs) responses to VEGF such as differentiation, proliferation, migration, and formation of vascular tubes [9, 12]. Besides, VEGF-A family contains different subtypes but the role of VEGF_165_ is well characterized in the facilitation of angiogenic sprouting and endothelial cell tube formation by in vitro and in vivo studies [13, 14].

VEGFR-1 and 2 contain four main regions: (1) the extracellular ligand-binding domain, (2) a short transmembrane domain, (3) a tyrosine kinase domain, and (4) a carboxyl-terminal region. Additionally, there are 7 immunoglobulin-like (Ig-like) domains in the extracellular region [15]. The VEGF-A binding site is placed in the 2nd and 3rd Ig-like domains (domains II and III) in VEGFR-2 and the 2nd Ig-like domain (domain II) in VEGFR-1. The fourth to seventh Ig-like domains in these receptors have a key role in receptor dimerization and activation [16]. In a study done by Otrock et al., [17] it has been indicated that the binding of VEGF to the domains II-III of VEGFR2 promotes high-affinity binding of VEGF, and Ig-like III domain deletion of VEGFR2 reduces the binding affinity to more than 1000-folds. By binding VEGF-A to VEGFR2, the receptor undergoes dimerization and trans-phosphorylation. The dominant phosphorylation occurs on VEGFR2 in the tyrosine residues at positions of 1175 and 1214, which triggers the signaling cascade via Phosphoinositide 3-kinase (PI3K), Akt, phospholipase C (PLC), P38, Ras/mitogen-activated protein kinase (Ras/MAPK), and P42/44 MAPK [18, 19]. The angiogenic signaling is mainly produced from the ligand-activated VEGFR-2 following downstream tyrosine auto-phosphorylation sites. Moreover, it has been revealed that the phospholipase C- Protein Kinase C - Mitogen-activated Protein Kinase (PLCγ-PKC-MAPK) pathway is highly activated in VEGF-VEGFR-2 complex formation and used as a crucial signal for pro-angiogenic signaling [10]. STAT3 is activated upon VEGF stimulation of ECs in vitro and in vivo through VEGFR2-dependent and Src-dependent mechanisms that leads to Bcl-2 and pro-survival effects [20, 21]. In lymphangiogenesis and the early stage of embryogenesis, angiogenesis is frequently regulated by VEGF-C/D and its receptor, VEGFR-3 [22]. Upon stimulation with VEGF-C, the PKC and Ras pathway was activated for this process. Within the activation of ECs by VEGF, the expression of matrix metalloproteinases (MMPs) is upregulated to break-down the extracellular matrix (ECM) to permit the migration of ECs [23]. Co-existence and interaction between various angiogenic factors, cell surface receptors, and intracellular signaling components often result in increasing crosstalk between various signaling pathways. Such synergistic angiogenic activities in the tumor microenvironment may promote tumor growth, invasion, and metastasis [24]. Moreover, genetic instability of cancer cells often accumulates mutations of oncogenes and tumor suppressor genes leading to the upregulation of angiogenic factors. Besides, as the tumor grows larger, the core becomes hypoxic, resulting in hypoxia-induced upregulation of pro-angiogenic factors such as vascular endothelial growth factor receptor (VEGFR) 1–3 and stabilization of hypoxia-inducible factor-1α (HIF-1α) activity [25, 26]. Anti-angiogenic-based therapeutic agents are mostly categorized based on the ability of (i) inhibiting transcription and translation of specific angiogenic factors; (ii) functional neutralization of pro-angiogenic factors; and (iii) specific binding to the extracellular domain of VEGF receptor which can effectively block ligand-triggered angiogenic activity [27, 28]. Despite the successful development of these agents for the treatment of various types of cancers, the survival effects seem rather modest for most cancer types. Besides, a majority of cancer patients showed intrinsic resistant-development toward these anti-angiogenic drugs [29].

### Membrane‐anchored mucin (MUC1)

Membrane-anchored mucin (MUC1), also known as episialin, EMA (epithelial membrane antigen), PEM (polymorphic epithelial mucin), and CA-15–3 antigen, is the second out of 75 tumor-associated antigens [30, 31]. MUC1 is known as a heavily glycosylated type 1 transmembrane mucin that normally expressed at the apical side of luminal or glandular epithelial cells [32]. Alternative pre-mRNA splicing creates MUC1 variants A, B, C, D, X, Y, Z, SEC, and REP. In a study done by Obermair et al. [33] it has been reported that primary ovarian cancer was negative for variant SEC but positive for variant REP. Moreover, expression of MUC1/SEC is related to the absence of malignancy whereas the expression of MUC1 splice variants A, D, X, Y, Z, and REP is linked to the incidence of malignancy [34].

MUC1 expressed in normal cells and tumor-associated MUC1 (TA-MUC1) represents many differences in their cellular distribution, biochemical features, and function [30]. Transmembrane MUC1 is also engaged in transducing cellular signaling cascades through interacting with receptors for growth and differentiation in cancer cells. The cytoplasmic tail of MUC1 (MUC1-CT) is involved in many signaling cascades including those involved in tumor cell resistant-development, proliferation, and survival, as well as cell-cell and cell-matrix interactions. Moreover, MUC1-CT forms complexes with transcription factors and translocates into the nucleus, where can alternate transcription of specific target genes at the transcriptional and post-transcriptional levels [32]. A remarkable depolarized expression of MUC1 glycoprotein throughout the entire cell surface has been reported in a large number of carcinomas of the breast, ovary, rectum, pancreas, colon, and prostate [30].

**Fig. 1:**
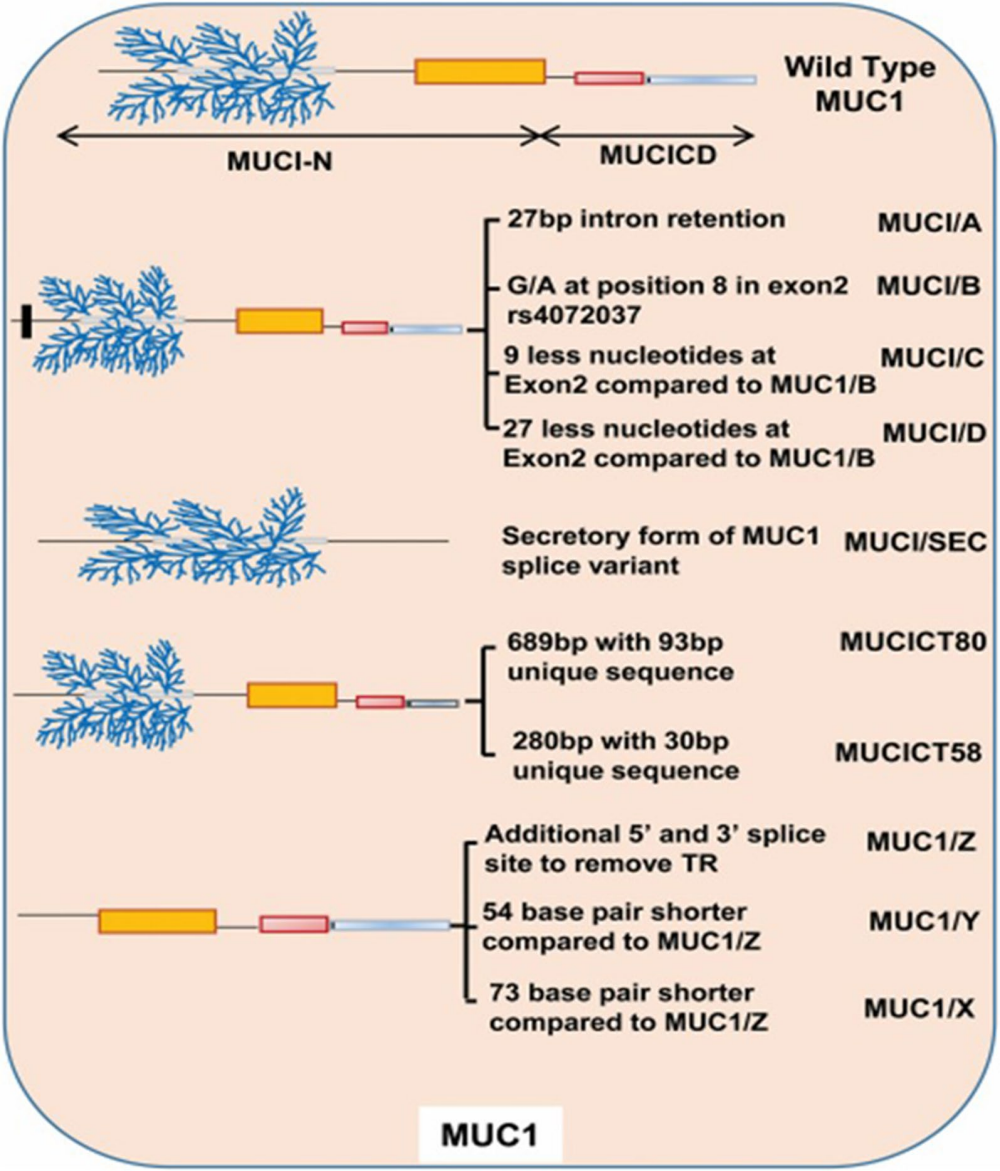
Different domains and different transcription variants of MUC1. Reprinted with permission from Ref. [97]

During stress conditions, MUC1 is auto-cleaved into MUC1-N, the longer N-terminal subunit, and MUC1-C, the shorter cytoplasmic-terminal subunit, resulting in the transduction of survival signaling [35]. These two subunits are connected via hydrogen bonds. The MUC1-N is containing PTS (proline, threonine, serine-rich) and SEA (Sperm protein, Enterokinase, and Agrin) domains. In PTS domain, the variable number tandem repeat (VNTR) region (a highly polymorphic exon) is responsible for the encoding of multiple 20–21 amino acid (aa) sequence repeats [36]. The structure of MUC1-C comprises a 58 aa extracellular domain (ECD), a 28 aa transmembrane domain (TMD), and a 72 aa cytoplasmic tail (CT) [37] (Fig. 1).

The cancer cells survive in a hypoxic environment by promoting pro-angiogenic gene expression and stimulating angiogenesis. In the hypoxic condition, the expression of pro-angiogenic factors is induced by MUC1. The increase of angiogenic factors including VEGF-A, connective tissue growth factor (CTGF), and platelet-derived growth factor-β (PDGF-β) subsequently results in tube formation in endothelial cells (ECs) and the creation of new blood vessels inside the tumor [38]. Scientists have indicated that overexpression of MUC1 in pancreatic and breast cancer cells can elevate synthesis and secretion of tumor angiogenic response, particularly VEGFs, via activation of intracellular signaling pathways such as Ras/mitogen-activated protein kinase (MAPK), Jak/Stat, and phosphoinositide 3-kinase (PI3K)/AKT/mechanistic target of rapamycin (PI3K/AKT/mTOR) [39, 40]. Additionally, the HIF-1α transcription factor binds to the MUC1 promoter and upregulates MUC1 expression [41]. Consistent with angiogenesis stimulation, MUC1-induced factors strengthen migratory and invasive properties of cancer cells [42, 43].

Although multiple targeted agents have been investigated for predicted pathways in angiogenesis, but still the majority of them have failed to achieve a satisfactory therapeutic outcome and have yet to be confirmed [44]. Identifying the correlation between aberrantly overexpressed MUC1 and angiogenetic-induced factors may represent a better comprehension in the development of novel therapeutic agents for cancer patients.

In this review, we aim to highlight the main molecular and cellular aspects of angiogenic possess in relation to the aberrant activation of MUC1 oncoprotein in tumor growth and metastasis. At the end, we will discuss the crosstalk between the pro-angiogenic factors like VEGF and aberrant cancer-associated signaling pathways to address the potential utility of targeting MUC1 oncoprotein in therapeutic strategies.

### Disruption of apoptotic response by MUC1-C

It has conclusively been shown that the apoptotic pathways have defected in numerous cancer cells, thereby many anticancer agents, which are designed to induce apoptosis, have failed clinically. MUC1 has been known to prevent the activation of the intrinsic apoptotic pathway in cancer cells. Studies have found that MUC1 overexpression restricts apoptosis via upregulating expression of the B cell lymphoma extra-large protein (Bcl-xL, an anti-apoptotic protein) and inactivating pro-apoptotic Bcl2-associated agonist of cell death (Bad) protein [24, 45]. Moreover, MUC1 overexpression reduces intracellular reactive oxygen species ROS levels which are elevated in hypoxic cells and activates apoptotic pathways through upregulating the expression of superoxide dismutase, glutathione peroxidase, and catalase [46, 47]. Additionally, MUC1 inhibits hypoxia-induced cell death and suppression of HIF-1α stability through reducing intracellular ROS and prolyl hydrolase-3 (PHD-3) activity in colon cancer cells [48]. Furthermore, ATP binding cassette (ABC) transporters evade the cancer cells from drug-induced cell death via efflux of anticancer drugs out of the cell [49]. This mechanism of resistance is mostly observed against amphipathic drugs. In a recent study, it has been reported that aberrantly overexpressed MUC1 increases resistant-development to the chemotherapeutic agent via interaction with multidrug resistance protein 1 (MRP1) [50].

### MUC1 signaling in tumor cell proliferation

Several kinds of research have demonstrated the crucial role of MUC1 in transcriptional regulation. As MUC1-CD localizes to the nucleus, it affects the gene transcription related to proliferation, apoptosis, angiogenesis, metastasis, drug resistance, and immune regulation [50–54]. In addition, MUC1-C activates several transcription factors such as STAT3, NF-κB, p53, and β-catenin through binding to the promoter region of the target genes and induces their aberrant expression in cancer cells [51, 55, 56]. Additionally, MUC1-C can promote loss of polarity with the induction of epithelial-mesenchymal transition (EMT) and interact with other cell surface molecules, including receptor tyrosine kinases (RTKs) [57].

Extensively branched Core 2 O*-*glycans were observed in normal cells. In contrast, in TA-MUC1, there are not Core 2 O*-*glycans due to the lack of Core 2 β6-GlcNAc-transferase activity. Core 1 O*-*glycans were extensively exhibited in MUC1-overexpressing breast cancer cells [58]. Furthermore, TA-MUC1 is highly sialylated, which causes an increase in the expression of α2, 3- and α2, 6-sialyltransferases in cancerous cells. Sialylation subsequently leads to truncation of sugar branches due to premature termination of chain elongation [59]. This hypoglycosylation uncovers the peptide core of TA-MUC1, MUC1-N cleavage, and diffusion by extracellular proteases. Moreover, the alteration of MUC1-N structure makes conformational changes in MUC1-C, which affects its ligand binding and triggers downstream cell signaling cascades such as Ras/Raf/MEK/ERK signaling pathway [60, 61]. Moreover, growth factors such as PDGF-α, PDGF-β, and CTGF are interceded by MUC1. These events mainly cause activation of MAPK and PI3K/Akt pathways, which potentiate the survival of tumor cells [43, 62, 63]. Hyperactivation of these critical signaling pathways has been extensively observed in MUC1-positive cancer cells including pancreas, breast, lung, and colon [50, 64].

With the stimulation of epidermal growth factor (EGF), MUC1-C directly binds to the epidermal growth factor receptor (EGFR) and translocates to the nucleus. After that, it binds to cyclin D1 (CCND1) and v-myb myeloblastosis viral oncogene homolog-like 2 (MYBL2) promoters and causes G1/S phase gene expressions such as thymidine kinase and dihydrofolate reductase [65–67]. Furthermore, Sahraei et al. [42] stated that the association of MUC1-CT with HIF-1α is simulated by PDGF-A and promotes proliferation and invasion of pancreatic ductal adenocarcinoma (PDA) cells. HIF-1α is a cancer hallmark that accelerates survival and proliferation under abnormal glucose metabolism. It regulates the metabolic pathways of proliferating cancer cells through the glycolytic pathway and enzyme expression. MUC1 is a modulator of hypoxic response through regulating the expression, stability, and activity of HIF-1α [68]. Furthermore, the interaction between MUC1 and HIF-1α leads to their stabilization and binding to multiple glycolytic gene promoters to enhance their expression in a hypoxia-dependent manner. It has been remarkably reported that MUC1 facilitates a higher expression of glucose uptake and gene metabolism in pancreatic cancer models [68]. Thereby, TA-MUC1 can facilitate cancer cell growth and survival via upregulating glucose uptake and metabolism [30].

### MUC1 in invasion and metastasis of different carcinomas

**Fig. 2:**
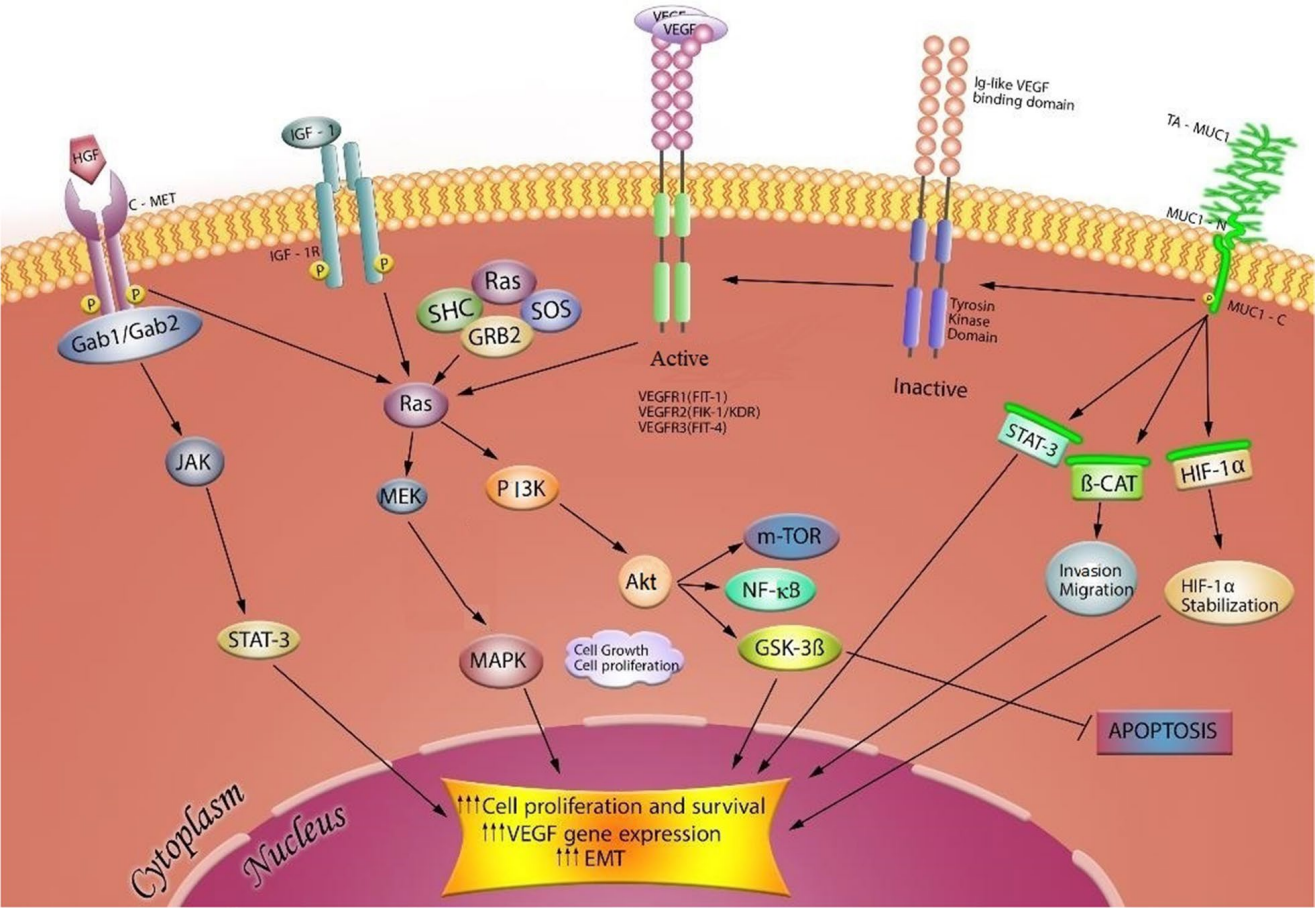
VEGF signaling pathways involved in angiogenesis and its crosstalk with TA-MUC1 in cancer cells. In this proposed model, activation of VEGF signaling and several MUC1-C activities in association with cancer have been illustrated. MUC1 overexpression is existing on the surface of a majority of cancer cells. The phosphorylation of its cytoplasmic domain is capable of translocation and interacting with pro-angiogenic and proliferative regulators such as HIF-1α, β-catenin, and STAT-3 which leading to the upregulation of target-gene expression and survival of cancer cells. In the case of resistance to angiogenesis inhibitors such as TKIs, MUC1 leads to sustained downstream signaling. Activation and auto-phosphorylation of VEGF family receptor, even in the presence of therapeutic agents and aberrant activation of the PI3K/AKT/mTOR, MAPK, and JAK/STAT3 in tumor progression, leads to the elevation of pro-angiogenic gene expression and migration as well as inhibition of GSK3 and thereby blocking the apoptosis. Moreover, MUC1 induces proliferative signaling through making interaction with tyrosine kinase receptor IGF-1R and IGF-1 mediated induction of VEGF. HGF regulates VEGF expression via the tyrosine-protein kinase c-Met receptor downstream pathways, PI3K/Akt, MAPK, and STAT3 in cancer cells

An essential step to tumor cell survival is metastasis. This multistep process allows cancer cells to detach from the basement membrane, degrade the adjacent matrix, attack the adjacent tissues, and/or arrive at the bloodstream. A description of the biological process by which cancer cells acquire their invasive behavior is EMT [24, 69]. Based on the available evidence, it has been concluded that MUC1-CT in association with β-catenin, translocates to the nucleus, represses E-cadherin expression, and disrupts cell adhesion. Moreover, the stabilized β-catenin/MUC1-C complex leads to upregulation of gene transcription expression of the EMT inducers Snail, Slug, vimentin, and Twist (Fig. 2) [40]. Previous findings have reported that MUC1 prompts EMT at the post-transcriptional level by controlling miRNAs which modulate EMT-related gene expression. The obtained results revealed that MUC1-C overexpression promoted adherent-junctions’ disruption and cytoskeleton rearrangement. Therefore, MUC1-C reduces the contact between cancer cells and enables basement membrane invasion [30]. Besides, the nuclear localization of the MUC1-CT/β catenin transcriptional complex induces the stimulation of PDGF-B. This event increases the invasive potential of PDA cells (39). Additionally, Cascio et al., [70] extended their work to the association of MUC1 with CIN85 (Cbl-interacting a protein of 85 kDa) which makes the invadopodia-like structures and aides breast cancer cell invasion. Such mechanisms describe that the overexpression of MUC1 leads to poor prognosis and metastasis in breast, pancreas, and colon cancer patients [71, 72]. Furthermore, several studies have described that as a ligand, TA-MUC1 binds to cell adhesion molecules such as selectins and intercellular adhesion molecule-1 (I-CAMs). This behavior of TA-MUC1 aids adherence of MUC1-expressing circulating tumor cells (CTCs) to a simulated blood vessel wall, then disseminating at distant sites to initiate secondary tumor formation [73]. Selectin binds to the carbohydrate epitope sLe^X^ (sialyl-Lewis x) and then increases sLe^X^ expression in MUC1-overexpressing colon cancer cells that correlated with metastasis [74, 75]. In melanoma, overexpression of MUC1 intervenes with integrin-mediated cell adhesion to the extracellular matrix and aggregates cancer cell invasiveness [76].

### Enhancement of tumor angiogenic response by MUC1

Cancer cells adapt to survive in a hypoxic environment by inducing the expression of proangiogenic factors and angiogenesis. Recent findings demonstrate that under hypoxic conditions, MUC1 elevates expression of proangiogenic gene levels such as CTGF, PDGF-B, and VEGF-A that, in turn, promotes the synthesis of new blood vessels within the tumor and tube formation within ECs. Based on the evidence, aberrantly overexpression of MUC1 in breast and pancreatic cancer cells drives synthesis and secretion of VEGF. Besides, MUC1 induces the expression of PDGF-A in cancer cells in association with HIF-1α. Interestingly, *MUC1* gene expression is regulated by HIF-1α, which binds to the MUC1 promoter region and modulates its expression. Furthermore, MUC1-CD induces *CTGF* gene expression, a potent mediator of metastasis and angiogenesis, via binding to its promoter region through β-catenin and p53. Therefore, it necessitates noting that such MUC1-induced factors not only promote angiogenic events but also stimulate the migratory and invasive features of cancer cells.

### Relationship of MUC1 with VEGF/VEGFR and IGF-1/IGF-1R signaling pathways

Woo et al. have reported that overexpression of MUC1 promoted IGF-1-IGF-1R/Akt/VEGF signaling in the human breast carcinoma cases [39]. Their findings have indicated that the expression of MUC1 stimulated the synthesis and secretion of VEGF through AKT and ERK1/2 signaling pathways. This VEGF production through MUC1 expression showed different effects includes; (1) VEGF overexpression by MUC1 transfection was observed in breast cancer cells, (2) MUC1 knocking down by MUC1 siRNA or AKT chemical inhibitor diminished MUC1-mediated VEGF induction, (3) The activation of insulin-like growth factor-1 receptor, which correlated with VEGF expression, occurred in MUC1-overexpressing cancer cells, (4) MUC1 overexpression augmented xenograft tumor growth in vivo when MDA-MB-231 human breast cancer cells were injected into NOD/SCID mice, (5) Increasing tumor growth and angiogenesis in a PyMT-MMTV/hMUC1 transgenic mouse model was observed by MUC1 overexpression, and (6) A high correlation between MUC1 and VEGF expression in human breast carcinoma was shown by the analysis of a human tissue microarray. The maximum level of VEGF is induced by Hypoxia. However, VEGF expression has also been reported in normoxia in many cell lines, which is a representative that other factors have a role in VEGF regulation. Regulation of VEGF expression is complex as its function is mainly controlled by several growth factors and cytokines including endothelial growth factor (EGF), IGF-1, PDGF, TGF-α, TGF-β, FGF, and interleukin-6 (IL-6) at various levels [77, 78]. Remarkably, the PI3K signaling pathway, via activation of protein kinase C (PKc), causes the driving of VEGF transcription under normoxic conditions in human fibrosarcomas and renal cell carcinomas [39].

Pro-angiogenesis gene expressions like *VEGF* gene are upregulated by phosphorylated AKT and ERK1/2 that subsequently stimulate ECs proliferation, migration, and tumor angiogenesis [79]. Additionally, Woo’s results showed diverse effects of AKT and ERK1/2 on VEGF expression level [39]. To determine whether AKT pathway has an inducing role in VEGF expression in MUC1-overexpression cancer cells, the expression of VEGF was inhibited by LY-294,002 (AKT inhibitor). Furthermore, VEGF suppression by an ERK1/2 inhibitor (PD-98,059) in the MUC1-overexpressing cancer cells illustrated significant results in the regulation of VEGF expression and these findings show exclusive roles for AKT and ERK1/2 signaling cascades in tumor survival by MUC1 [39]. Besides, their study demonstrated that the MUC1-mediated signaling pathway prompts VEGF expression in association with activation and phosphorylation of IGF-1R. IGF-1 increases VEGF expression in cancer cells through binding to its receptor promotes angiogenesis [80, 81]. Moreover, PI3K/AKT and ERK1/2 pathways regulate the functional effect of IGF-1R on angiogenesis progression (Fig. 2) [82, 83]. The interaction between MUC1 and IGF-1R causes IGF-1R to become a powerful angiogenic factor in cancer cells [78, 84]. Furthermore, MUC1 overexpression can directly affect the activation of IGF-1R. For determining the MUC1 and IGF-1R relationship, Woo and his colleagues conducted three experiments and concluded that MUC1 extensively increases the phosphorylation of IGF-1R [39]. Moreover, inhibiting MUC1 expression by siRNA transfection of MDA-MB-231 clones abolished IGF-1R phosphorylation. In vivo studies have also illustrated a significant increase in the IGF-1R phosphorylation compared to the control cells in MUC1-expressing murine breast cancer cells. As a result, controlling IGF-1R signaling pathways by antagonizing MUC1 could be a critical step during tumor development. In brief, their study revealed that MUC1 expression induced the phosphorylation of IGF-1R and AKT. They showed that VEGF is a downstream component of the MUC1/IGF-1R/AKT cascade and its activation plays an important role in tumor angiogenesis associated with MUC1 [39]. Thus, as depicted in Fig. 2, a remarkable correlation between overexpression of MUC1 and VEGF expression has been identified in many cancers and upregulation of PI3K/AKT signaling mainly contributes to the activation of diverse effectors to promote tumor growth and survival [85]. MUC1 induces PI3K/AKT pathway which can lead to VEGF secretion through both HIF-1α dependent and independent mechanisms. The PI3K/AKT signaling also induces the expression of other pro-angiogenic factors such as nitric oxide and angiopoietins. In addition, STAT3 phosphorylation shows a significant role in VEGF and PI3K/AKT-mediated HIF-1α expression [85]. According to this evidence, it has been suggested that binding of STAT3 and HIF-1α to the VEGF promoter is essential for maximum transcription of VEGF mRNA in hypoxic conditions [86].

In one study, Giatromanolaki et al. investigated coexpression of MUC1 glycoprotein with Multiple angiogenic factors such as vascular endothelial growth factor (VEGF), VEGF-receptor, basic fibroblast growth factor (bFGF), and bFGF-receptor (FGFR-2) in non-small cell lung cancer. Their results displayed significant coexpression of MUC1 with VEGF [87]. In another study, Rosa et al. studied immunostains for D2-40, CD31, CD34, vascular endothelial growth factor A (VEGF-A), and mucin 1 (MUC-1) in invasive micropapillary colorectal carcinoma. They observed strong coexpression of VEGF-A and CD31 in the tumor cells [88]. These findings also confirmed by Xu`s study, in which the inhibition of MUC-1 by a specific siRNA resulted in a notable decrease of VEGF expression in non‑small cell lung cancer cell lines [89] (Table 1).

**Table 1:** List of studies on the cross-talk between MUC1 and VEGFR proteins in various cancers

Cross-talk evidence	Cancer type	References
Stimulation of synthesis and secretion of VEGF through AKT and ERK1/2 signaling pathways by MUC1 expression	Human breast carcinoma	[39]
Induction of NRP1-dependent VEGFR signaling and angiogenesis by modulating TA-MUC1	Pancreatic ductal adenocarcinoma cells	[96]
Strong co-expression of VEGF-A and MUC-1 in neoangiogenesis of Invasive micropapillary carcinoma cells	Invasive micropapillary colorectal carcinoma	[88]
MUC1 overexpression was frequent in cases with up-regulated VEGF/KDR angiogenic pathway in the sample patients	Non-small cell lung cancer	[87]
Co-transcription of MUC1 and VEGF mRNA in blood samples of patients with advanced non-small cell lung cancer	Non-small cell lung cancer	[98]
Increasing VEGF expression in MUC1-overexpressing NSCLC cells through phosphorylated Akt and ERK1/2 pathways	Non-small-cell lung cancer cells	[99]
Decreasing expression of VEGF by MUC-1 inhibition	Non-small-cell lung cancer cells	[89]

### Neuropilin-1 induces VEGF signaling and angiogenesis via MUC1 signaling

Neuropilins, NRP-1, and NRP-2 are transmembrane receptors that lack the tyrosine kinase domain and are co-receptors for the VEGF family. The VEGF_165_ subtype can simultaneously bind to both NPR-1 and VEGFR2 [90]. Studies have suggested that NRP-1 enhances the binding of VEGF_165_ and stimulates angiogenesis via linking NRP-1 and VEGFR2 [91]. It has also been reported that the neutralization of VEGF and NRP-1 suppresses vascular remodeling and tumor growth [92]. Overexpression of NRPs in breast, prostate, colon, and lung carcinomas is associated with disease progression, poor prognosis, and metastasis [93, 94]. Based on the available evidence, there are three ways that VEGF signaling is upregulated by NRP-1 in endothelial and tumor cells: (1) autocrine, (2) paracrine, and (3) juxtacrine. Autocrine signaling inhibits tumor cell apoptosis and paracrine signaling induces angiogenic progression from tumor cells to ECs. In juxtacrine signaling, at the same time, VEGF binds to VEGFR2 on ECs and to NRP-1 on tumor cells to induce angiogenesis and tumor growth, respectively [91, 95].

So far, TA-MUC1 has been well-characterized to have a potential role in EMT stimulation, pro-angiogenic, and pro-metastatic proteins expression in cancer cells, as well as resistant-development to current anti-cancer therapeutic agents [40, 42, 50]. In a study, Zhou et al., reported an NRP1-dependent VEGFR signaling by modulating TA-MUC1 in PDA cells [96]. They showed that overexpression of MUC1 in over 80% of PDA patients and its aberrant expression increases the levels of NRP-1 and VEGF, which subsequently causes a pro-angiogenic tumor microenvironment. Their data also demonstrated that there is a positive correlation between the expression of TA-MUC1 and NRP-1 levels in human cancer cells and mouse PDA xenograft model. TA-MUC1^high^/NRP-1^high^ /PDA cells facilitated the induction of ECs tube formation, the new blood vessel production, and increased metastasis in a zebrafish xenograft model. Simultaneously, in these TA-MUC1^high^/NRP-1^high^ PDA cells, high induction of the proteins related to EMT, such as N-cadherin and Vimentin, were observed. Thereby, according to their findings, blocking of NRP-1/VEGF signaling considerably reduced tube formation, new vessel generation, and metastasis prompted in MUC1-overexpressing PDA cells. Moreover, they observed a significant reduction in VEGFR signaling and tumor growth by blocking the interaction between NRP-1 and VEGF_165_ using an NRP-1 antagonist [96].

## Conclusions

In summary, due to multiple functions of MUC1 in cancer progression, it is essential to realize whether MUC1 plays an initiating role in metastasis and angiogenesis. Based on the available evidence, the elevated level of MUC1 is seem to be critical in cancer progression once an initiating oncogenic event has occurred. However, it can be reasonably inferred that MUC1 does not trigger malignant transformation but plays a significant role in generating vital conditions for cancer development. In this review, we discussed MUC1 influence on the angiogenic-development of cancers under hypoxic conditions or in making interaction with pro-angiogenic factors such as VEGF, IGF, and related intracellular signaling cascades. Among MUC1 functions discussed here, it is rather required more investigation into the finding of which oncogenic pathway plays initiating roles in angiogenesis, and which fosters involves in invasion, proliferation, and metastasis of cancer cells in response to aberrantly overexpressed MUC1. Besides, antagonizing TA-MUC1 and VEGF expression together could be used as a biomarker to predict the treatment efficacy of currently existing therapeutic agents in cancer patients as well as novel approaches of cancer targeted-therapy.

## Data Availability

Not applicable.

## Abbreviations

ABC: ATP binding cassette
AMOP: Other protein
Bcl-xL: B cell lymphoma extra-large
β-CAT: β-catenin
CT: Cytoplasmic tail
CA-15–3: Cancer antigen 15−3
CTCs: Circulating tumor cells
CTGF: Connective tissue growth factor
ECD: Extracellular domain
ECs: Endothelial cells
EGF: Endothelial growth factor
EGF: Epidermal growth factor
EMT: Epithelial to mesenchymal transition
ERK: Extracellular signal-regulated kinase
FGF: Fibroblast growth factor
HGF: Hepatocyte growth factor
HIF-1α: Hypoxia-inducible factor 1-alpha
I-CAMs: Intercellular adhesion molecule-1
IGF-1: Insulin-like growth factor-1
IGF-1R: Insulin-like growth factor-1receptor
IL: Interleukin
MAPK: Mitogen-activated protein kinase
MMP: Matrix metalloproteinase
MRP1: Multidrug resistance protein 1
MUC1: Mucin 1
NRP-1: Neuropilins
NIDO: Nidogen-like domain
NOD/SCID: Nonobese diabetic/severe combined immunodeficiency
PDA: Pancreatic ductal adenocarcinoma
PDGF: Platelet-derived endothelial growth factor
PDGF-β: Platelet-derived growth factor-β
PLC: Phospholipase C
PI3K: Phosphoinositide 3-kinase
PKc: Protein kinase C
PTS: Proline, threonine, serine
ROS: Reactive oxygen species
RTK: Receptor tyrosine kinase
STAT3: Signal transducer and activator of transcription 3
SEA: Sperm protein enterokinase agrin
TA-MUC1: Tumor associated MUC1
TGF: Transforming growth factor
TMD: Transmembrane domain
TNF-α: Tumor necrosis factor-alpha
VEGF: Vascular endothelial growth factor
VEGFR: Vascular endothelial growth factor receptor
VNTR: Variable number tandem repeat
